# Epigallocatechin Gallate Ameliorates Granulosa Cell Developmental via the Eukaryotic Initiation Factor 2 Alpha/Activating Transcription Factor 4 Pathway in Hyperthyroid Female Rats

**DOI:** 10.3390/antiox14091092

**Published:** 2025-09-06

**Authors:** Ying Sun, Mingqi Wu, Haoyuan Feng, Yilin Yao, Rui Chen, Yanzhou Yang, Cheng Zhang

**Affiliations:** 1 College of Life Science, Capital Normal University, Beijing 100048, China; 2220802055@cnu.edu.cn (Y.S.); 2220802057@cnu.edu.cn (M.W.); 2230802063@cnu.edu.cn (H.F.); 2230801011@cnu.edu.cn (Y.Y.); 2230802062@cnu.edu.cn (R.C.); 2 Key Laboratory of Fertility Preservation and Maintenance, Ministry of Education, Key Laboratory of Reproduction and Genetics in Ningxia, School of Basic Medicine, Ningxia Medical University, Yinchuan 750004, China

**Keywords:** hyperthyroidism, ovary, GRP78, OS, ERS, EGCG

## Abstract

Follicular development is recognized as a highly complex biological process regulated by multiple factors. Thyroid hormone (TH) is considered one of the key regulators of female reproduction, and its dysregulation can significantly impair follicular development. Epigallocatechin gallate (EGCG), the main active component of green tea, possesses strong antioxidant properties. Numerous studies have demonstrated that EGCG positively influences reproductive function in both humans and animals. However, whether EGCG directly affects follicular development under conditions of TH dysregulation remains poorly understood. The primary objective of this study was to investigate the impact of hyperthyroidism on ovarian development, examine whether EGCG could mitigate the adverse effects of TH dysregulation, and elucidate the underlying molecular mechanisms. In the T_4_-induced hyperthyroidism rat model, ovarian tissues were serially sectioned for Hematoxylin-Eosin (HE) and Masson’s trichrome staining to assess morphological changes, and follicle numbers were quantified at each developmental stage. Granulosa cell (GC) viability, proliferation, and apoptosis induced by T_3_ were evaluated using CCK8, EdU, and TUNEL assays, respectively. Antioxidant enzyme activity was measured, and the expression levels of related proteins were analyzed via Western blotting. Results showed that hyperthyroidism altered ovarian structure, significantly increasing the number of atretic follicles. Levels of antioxidant enzymes, including Superoxide Dismutase (SOD), Glutathione Peroxidase (GSH-PX), and Catalase (CAT), were markedly decreased, whereas the lipid peroxidation product malondialdehyde (MDA) was significantly elevated. Furthermore, all ERS-related proteins, phosphorylated Eukaryotic Initiation Factor 2 Alpha (p-eIF2α), Activating Transcription Factor 4 (ATF4), C/EBP homologous protein (CHOP), and Caspase-3, were upregulated, accompanied by decreased glucose-regulated protein 78 (GRP78) expression. Treatment with EGCG alleviated these detrimental effects of hyperthyroidism. At the cellular level, high concentrations of T_3_ reduced GC viability and proliferation while increasing apoptosis. Reactive oxygen species levels were elevated, and GRP78 expression was decreased. Notably, all T_3_-induced effects were reversed by EGCG treatment. In summary, this study demonstrates that hyperthyroidism induces oxidative stress in GCs, which triggers endoplasmic reticulum stress via the eIF2α/ATF4 pathway and leads to apoptosis. EGCG mitigates apoptosis by enhancing antioxidant capacity, thereby preserving ovarian function. These findings establish EGCG as a protective agent for maintaining ovarian health and fertility.

## 1. Introduction

Ovarian follicular development is a highly complex biological process, with the majority of follicles undergoing progressive loss through follicular atresia [1]. Follicle development is regulated by numerous factors, including thyroid hormone (TH). When the balance between survival and cytotoxic factors is disrupted, follicular development is impaired. In hyperthyroid conditions, elevated TH levels can have detrimental effects on ovarian reproductive function [2]. Clinical observations indicate that hyperthyroid patients often exhibit increased luteinizing hormone levels during both the follicular and luteal phases [3], which may contribute to menstrual irregularities such as amenorrhea, oligomenorrhea, and anovulation [4]. However, the underlying mechanisms remain poorly understood.

Emerging evidence suggests that TH possesses pro-oxidant properties [5], and hyperthyroidism is commonly associated with disrupted redox homeostasis [6,7,8]. Excessive generation of reactive oxygen species (ROS) can overwhelm antioxidant defenses, leading to oxidative imbalance and triggering OS. Oxidative damage, primarily mediated by ROS, is counteracted by key antioxidant enzymes, including superoxide dismutase (SOD), catalase (CAT), and glutathione peroxidase (GSH-PX) [9]. Malondialdehyde (MDA), the end product of lipid peroxidation, accumulates during oxidative insult and is widely recognized as a reliable biomarker for assessing the severity of oxidative damage [10].

Hyperthyroidism-induced oxidative stress (OS) leads to oxidative damage in intracellular proteins, resulting in the accumulation of unfolded proteins [11]. When these unfolded proteins excessively accumulate in the endoplasmic reticulum (ER), they bind to the molecular chaperone glucose-regulated protein 78 (GRP78) within the ER lumen. Upregulation of GRP78 triggers its dissociation from the Protein Kinase R-like Endoplasmic Reticulum Kinase (PERK) complex on the ER membrane. This dissociation allows PERK to become phosphorylated (p-PERK), thereby activating the PERK-eIF2α signaling pathway. Under conditions of persistent ER stress, phosphorylated eIF2α (p-eIF2α) selectively induces the transcription factor activating transcription factor 4 (ATF4). ATF4 then translocates into the nucleus to regulate the transcription of its downstream target gene, C/EBP homologous protein (CHOP) [12], ultimately activating cysteine-aspartic acid protease 3 (Caspase-3) and inducing apoptosis [13]. However, the regulatory relationship between OS and ER stress in ovarian GCs remains to be fully elucidated.

Epigallocatechin gallate (EGCG), the main active component of green tea, is a potent natural antioxidant with strong free radical scavenging capacity [14]. EGCG directly neutralizes free radicals and reduces lipid peroxidation, including the formation of MDA. It also enhances the activity of antioxidant enzymes, such as T-SOD, CAT, and GSH-PX, through the Nrf2/Keap1 pathway [15,16]. Furthermore, EGCG modulates TH levels by inhibiting thyroid peroxidase (TPO) activity, thereby suppressing TH synthesis [17]. Studies have also demonstrated that EGCG inhibits ROS production and protects ARPE-19 cells from apoptosis [18]. In oocytes exposed to methylparaben (MP), increased ROS impairs maturation, whereas EGCG exerts protective effects by reducing both ROS levels and ERS [19].

Although the antioxidative effects of EGCG against OS and endoplasmic reticulum stress (ERS) are well documented, its ability to preserve follicular development under hyperthyroid conditions, where accelerated follicular atresia occurs, remains unclear. We hypothesize that EGCG may mitigate hyperthyroidism-induced abnormalities in follicular development through its potent antioxidant properties.

The present study was designed to systematically investigate the detrimental effects of hyperthyroidism on follicular development and the underlying molecular mechanisms while simultaneously evaluating the therapeutic potential of EGCG in mitigating TH dysregulation-induced impairment of follicular development.

In the present study, we demonstrated that hyperthyroidism induces OS, which subsequently triggers ERS via the GRP78/eIF2α/ATF4 pathway, ultimately promoting granulosa cell (GC) apoptosis. Notably, these effects were alleviated by EGCG, which enhanced antioxidant capacity and downregulated ERS.

## 2. Materials and Methods

### 2.1. Reagents and Antibodies

Unless otherwise specified, most reagents and chemicals used in the present study were purchased from Sigma-Aldrich (St. Louis, MO, USA). Rabbit polyclonal anti-GRP78 (ab21685) was purchased from Abcam (Cambridge, MA, USA). Mouse monoclonal anti-ATF4 (sc-390063) was purchased from Santa Cruz Biotechnology, Inc. (Dallas, TX, USA). Rabbit monoclonal anti-p-eIF2α (#D9G8) and rabbit monoclonal anti-eIF2α (#D7D3) were obtained from Cell Signaling Technology, Inc. (Danvers, MA, USA). Rabbit monoclonal anti-CHOP (T56694S), and rabbit monoclonal anti-Caspase-3 (T40044F) were purchased from Abmart Shanghai Co., Ltd. (Shanghai, China). Rabbit polyclonal anti-β-actin (BE39995), horseradish peroxidase (HRP)-conjugated anti-rabbit, and anti-mouse IgG were from Bio-easy (Beijing, China). Medium 199 (M199) was purchased from Biological Industries (Beit Haemek, Israel). Fetal bovine serum was from PAN-Biotech (Aidenbach, Bavaria, Germany). Penicillin and streptomycin were purchased from Invitrogen (Carlsbad, CA, USA). The enhanced chemiluminescence (ECL) detection kit was obtained from LABLEAD (Beijing, China).

### 2.2. Animal Treatments

The 21-day-old female Sprague-Dawley (SD) rats used in the experiment were purchased from Beijing Vital Laboratory Animal Technology Co., Ltd. (Beijing, China). All experiments were conducted at the Animal Experiment Center of Capital Normal University (Beijing, China). The rats were maintained under constant temperature (24–26 °C) and humidity (60% ± 2%) with a 12/12-h light/dark cycle.

Hyperthyroidism was induced in 25 rats via daily subcutaneous injections of L-thyroxine (T_4_; Sigma-Aldrich, HY-18341) at 250 μg/kg body weight for two weeks. The T_4_ was dissolved in saline containing 0.025% NH_4_OH/methanol [20]. Subsequently, hyperthyroid rats were randomly divided into five groups (*n* = 5/group) and treated with EGCG (0, 10, 50, 100, or 200 mg/kg/day, dissolved in physiological saline) via intraperitoneal injection for an additional two weeks while continuing T_4_ administration [21,22]. Control rats (n = 5) received subcutaneous injections of the solvent vehicle (physiological saline containing 0.025% NH_4_OH/methanol) in equivalent volumes for 4 weeks, with physiological saline alone being administered during the final two weeks.

All animals were euthanized via carbon dioxide (CO_2_) inhalation at the end of the experiment. CO_2_ was delivered at 20–30% of the chamber volume per minute to avoid distress, with 100% CO_2_ maintained. Experimental protocols were approved by the Institutional Animal Care and Use Committee of Capital Normal University (Approval No.:2022025) and complied with the Guide for the Care and Use of Laboratory Animals (National Research Council, 2011) and China Animal Welfare Guidelines.

### 2.3. Granulosa Cell Isolation and Culture

Granulosa cell isolation was performed as described previously [23]. Briefly, ovaries were mechanically disrupted using a 1 mL syringe to release granulosa cells into culture medium (M199 supplemented with 10% fetal bovine serum and 1% penicillin-streptomycin). The cell suspension was filtered through a 40-μm nylon mesh filter and centrifuged at 1500 rpm for 15 min to pellet cells. After supernatant removal, cells were resuspended in fresh culture medium and seeded into pre-equilibrated culture dishes. Cells were maintained in a humidified incubator (5% CO_2_, 37 °C) for subsequent experiments.

### 2.4. Protein Extraction and Western Blotting

Ovaries were dissected free of adipose tissue and rinsed with ice-cold 1× PBS. After blot-drying, tissues were minced into fragments and transferred to pre-chilled 1.5 mL microcentrifuge tubes. 200 μL of ice-cold prepared cell lysis solution (containing 1× phosphatase inhibitor cocktail and 1× protease inhibitor cocktail) was added. Tissues were homogenized using the Polytron homogenizer (30 s pulses × 3) followed by vortexing at 4 °C for 30 min. Lysates were centrifuged at 13,600× *g* for 10 min at 4 °C. Supernatants were collected as protein extracts. For granulosa cells, cells in 6-well plates were washed thrice with 1× PBS, then lysed on ice with 100 μL of prepared cell lysis solution. Cell monolayers were scraped using a cell lifter, and lysates were processed as described for ovarian tissue.

Protein concentrations were quantified using a BCA assay kit (Beyotime Biotechnology, Shanghai, China, Cat# P0012). Equal amounts of protein (20 μg/lane) were denatured in Laemmli buffer at 95 °C for 5 min, separated by SDS-PAGE (S1:80 V/40 min, S2:120 V/1 h) [24], and transferred onto PVDF membranes (0.45 μm; Bio-Rad, CA, USA, Cat# 1620177). Membranes were blocked with 5% (*w*/*v*) BSA in PBST for 1 h at room temperature (RT), then incubated overnight at 4 °C with the following primary antibodies: rabbit anti-GRP78 (1:1000), mouse anti-ATF4 (1:500), rabbit anti-p-eIF2α (Ser51) (1:1000), rabbit anti-eIF2α (1:1000), rabbit anti-CHOP (1:1000), rabbit anti Caspase-3 (1:1000), rabbit anti-MMP9 (1:500), and rabbit anti-β-actin (1:1000); after six 5-min washes with PBST, membranes were incubated with HRP-conjugated secondary antibodies (1:2000–1:5000) for 2 h at RT. Following an additional six 5-min washes with PBST, signals were developed using ECL Prime and captured on an ImageQuant LAS 4000 system. Band intensities were quantified with AlphaEaseFC 4.0 software.

### 2.5. Hematoxylin-Eosin (HE) Staining

As described before, rat ovarian tissue was subjected to conventional dehydration followed by paraffin embedding and sectioning (6 μm thick) after fixation in paraformaldehyde. The paraffin sections were subjected to gradient rehydration treatment, stained with hematoxylin for 3 min, rinsed with running water (1 min), differentiated with 1% hydrochloric acid (10 s), rinsed again (1 min), counterstained with eosin (1 min), and finally rinsed (1 min). After gradient dehydration and clearing, the sections were mounted using neutral resin (Solarbio Science & Technology, Beijing, China).

### 2.6. Follicle Counting Methodology

For quantitative analysis, every fifth serial ovarian section was systematically evaluated to avoid overcounting. Follicles were classified according to strict morphological criteria: primordial follicles (smallest structures with a single layer of flattened granulosa cells and no zona pellucida), primary follicles (cuboidal granulosa cells with visible zona pellucida but no antrum), secondary follicles (multiple granulosa cell layers with emerging antral spaces and oocyte embedded in cumulus oophorus), tertiary follicles (largest structures with dominant antral cavities and well-defined cumulus-oocyte complexes), and atretic follicles (characterized by pyknotic oocyte nuclei, disorganized granulosa cells, and fragmented zona pellucida). Primordial and primary follicles were counted whenever encountered, while secondary and tertiary follicles were only tallied when the oocyte nucleus was visible to prevent duplicate counts of large follicles across sequential sections [24,25].

### 2.7. Masson Trichrome Staining

Following standardized dehydration processing, paraffin-embedded tissue sections underwent histological staining utilizing a commercial Masson’s Trichrome Stain Kit (Sigma-Aldrich, St. Louis, MO, USA) in strict accordance with the manufacturer’s protocol. The staining regimen comprised the sequential application of: (1) Weigert’s iron hematoxylin for nuclear staining (5 min), followed by thorough rinsing under running tap water; (2) a 1% Biebrich scarlet-acid fuchsin solution for staining of muscle fibers and cytoplasm (5 min), succeeded by a distilled water wash; (3) treatment with 1% phosphomolybdic acid (5 min) immediately preceding direct staining of collagen fibers with 2.5% aniline blue (5 min), after which a brief rinse was applied; and (4) a final differentiation step employing 1% glacial acetic acid (1 min). Histological images were acquired using an optical microscope (ECLIPSE Ci-L, Nikon, Tokyo, Japan) equipped with a digital camera.

### 2.8. Quantification of Oxidative Stress Markers

Superoxide dismutase (SOD), glutathione peroxidase (GSH-PX), catalase (CAT), and malondialdehyde (MDA) levels were assessed in ovarian tissue homogenates. Tissues were homogenized in ice-cold physiological saline (1:9, *w*/*v*) and centrifuged at 3000× *g* for 10 min at 4 °C. The supernatant was collected, and total protein concentration was quantified using the bicinchoninic acid (BCA) assay kit (Nanjing Jiancheng Bioengineering Institute, Nanjing, China).

#### 2.8.1. SOD Activity Assay

The reaction mixture consisted of 0.2 mL assay buffer, 0.02 mL sample supernatant, and 0.2 mL enzyme-substrate solution. Following incubation at 37 °C for 40 min, 2 mL chromogenic agent was added. After a 10-min reaction period at ambient temperature, absorbance was measured at 550 nm using a microplate reader.

#### 2.8.2. GSH-PX Activity Assay

The reaction system contained 1 mL buffer, 0.2 mL reduced glutathione (GSH), and 0.2 mL sample supernatant. After pre-warming at 37 °C for 5 min, the reaction was initiated by adding 0.1 mL hydrogen peroxide (H_2_O_2_). Termination was achieved after 3 min with 0.5 mL precipitating agent, followed by centrifugation. Subsequently, 1 mL supernatant was combined with 0.25 mL chromogen, and absorbance was recorded at 412 nm.

#### 2.8.3. CAT Activity Assay

A mixture of 0.1 mL sample supernatant and 1 mL buffer was pre-incubated at 37 °C. The reaction was initiated with 0.1 mL H_2_O_2_ and terminated after 60 s with 1 mL ammonium molybdate solution. Absorbance was measured immediately at 405 nm.

#### 2.8.4. MDA Content Determination

Sample supernatant (0.1 mL) was mixed with 0.2 mL assay buffer and 0.2 mL thiobarbituric acid (TBA) reagent. The mixture was heated at 95 °C for 40 min, cooled to room temperature, and centrifuged at 3000× *g* for 10 min. Absorbance of the supernatant was measured at 532 nm.

For all assays, blank and standard (calibrator) controls were processed concurrently with experimental samples. Absorbance values were recorded using a microplate reader, and enzymatic activities/MDA concentrations were normalized to total protein content according to the manufacturer’s protocols (Nanjing Jiancheng Bioengineering Institute, Nanjing, China).

### 2.9. ROS Detection

The cells in the glass-bottom confocal dishes were washed thrice with 1× PBS (pH 7.4). The DCFH-DA (Lablead, Beijing, China) probe was diluted in serum-free medium to a final concentration of 10 μM. Cells were loaded with the probe and incubated at 37 °C in the dark for 30 min. The cells were examined and photographed under a fluorescence microscope LSM 780 (Zeiss, Jena, Germany) using a 488 nm excitation wavelength and a 525 nm emission wavelength. Fluorescence intensity was quantified using ImageJ 1.53e.

### 2.10. Analysis of Cell Viability

Cell activity was assessed using a CCK-8 Kit (Dojindo, Kumamoto, Japan). The cells cultured in 96-well flat-bottom plates were incubated with 10 μL CCK-8 reagent at 37 °C for 2 h, and the optical density value was measured at 450 nm with a microplate reader. Cell viability was calculated based on the OD value.

### 2.11. EdU Detection

According to the instructions of the EdU kit (Beyotime Biotechnology, Shanghai, China), cell proliferation was detected. The cells cultured in glass-bottom culture dishes were incubated with 50 μM EdU for 2 h, then washed three times with 1× PBS. The cells were fixed with 4% paraformaldehyde at room temperature for 0.5 h and permeabilized with 0.5% Triton X-100 for 10 min, followed by EdU staining. The cell nuclei were counterstained with Hoechst 33, 342 for 0.5 h. The cells were examined and photographed under a fluorescence microscope using a maximum excitation wavelength of 346 nm and a maximum emission wavelength of 460 nm.

### 2.12. TUNEL Assay

The TUNEL cell apoptosis detection kit (KeyGEN, Beijing, China) was used to detect the level of cell apoptosis. Cells cultured in glass-bottomed culture dishes were fixed with 4% paraformaldehyde at room temperature for 30 s, followed by permeabilization with 1% Triton X-100 for 5 min. After washing with 1× PBS three times, TdT buffer was added to the culture dish and incubated at 37 °C for 1 h. Then, 50 μL of streptavidin-TRITC labeled solution was added to each well and incubated at 37 °C in the dark for 30 min. The cell nuclei were stained with a diluted DAPI solution (1:200) at room temperature in the dark for 10 min. The cells were observed and photographed under a fluorescence microscope using the maximum excitation wavelength of 543 nm and the maximum emission wavelength of 571 nm.

### 2.13. Statistical Analysis

The experiments were repeated at least 3 times. All experimental data are presented as mean ± SEM. Data were analyzed using GraphPad Prism 8 software (GraphPad Software, La Jolla, CA, USA), and statistical differences between groups were calculated using one-way (repeated measures) ANOVA or two-way ANOVA. When significant differences were found, Bonferroni post hoc comparisons were used to compare the means. In addition, statistical significance was considered at *p* < 0.05.

## 3. Results

### 3.1. Effects of EGCG on the Ovarian Structure of Hyperthyroid Rats

**Figure 1:**
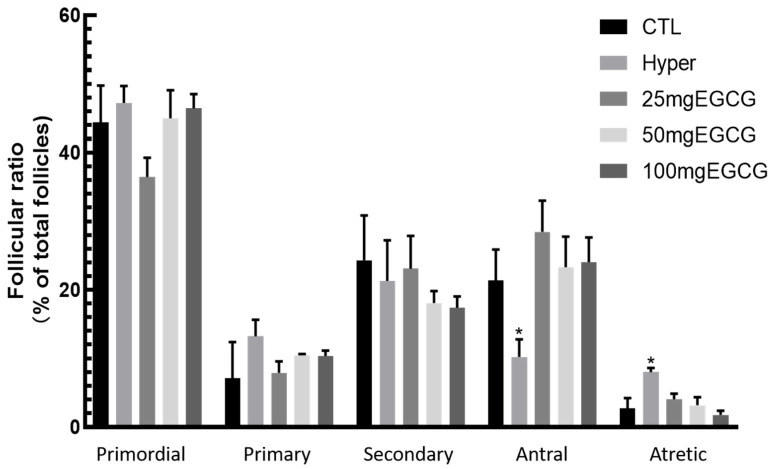
The effect of EGCG on the number of follicles at different stages in hyperthyroid rats. Establish a hyperthyroid rat model by intraperitoneal injection of L-thyroxine for 14 days, then treat with EGCG (25, 50, and 100 mg/kg body weight) by intraperitoneal injections. The numbers of different stage follicles were counted. * *p* < 0.05, compared with CTL.

**Figure 2:**
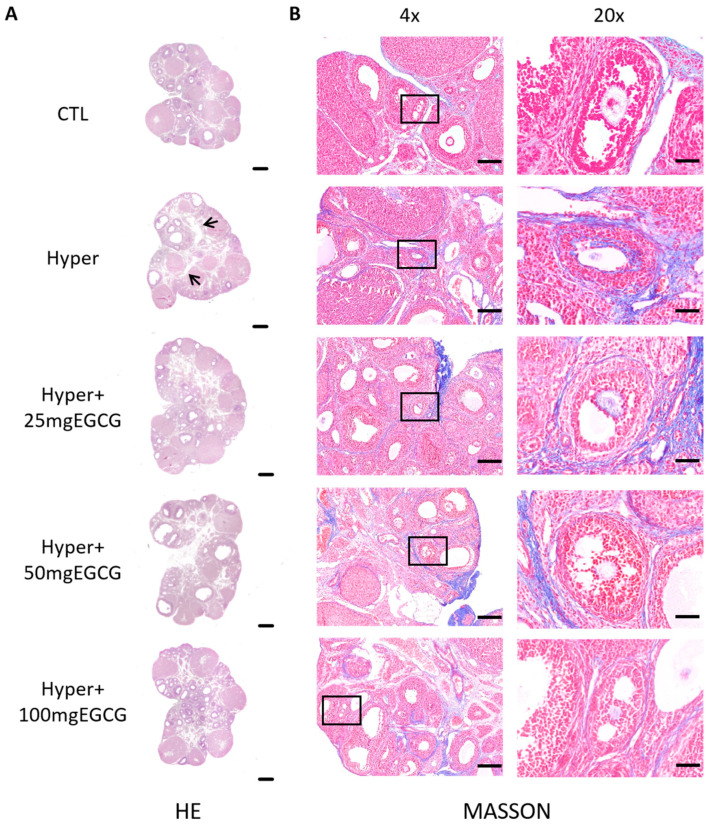
Effects of EGCG on the ovarian morphology and structure of hyperthyroid rats. The ovaries of different groups were collected, and the structure of ovaries were detected. (**A**) Representative H&E staining images of rat ovaries, scale bars = 500 μm, arrows indicate cavities; (**B**) Masson staining, blue represents collagen fibers, red denotes unstained areas, 4× scale bars = 20 μm, 20× scale bars = 50 μm.

To evaluate the effects of EGCG on hyperthyroid rat ovaries, hyperthyroid rats were treated with varying doses of EGCG. The 200 mg/kg EGCG group exhibited toxic symptoms, including anorexia, lethargy, and diarrhea on the first day of administration, followed by mortality on day 2, indicating that this dose exceeded the maximum safe limit. In the remaining groups, analysis of paraffin-embedded ovarian sections revealed that hyperthyroid rats had a significant increase in atretic follicle counts (*p* < 0.01) and a reduction in antral follicle numbers compared to controls (Figure 1). Histological examination further demonstrated a clear segregation between the cortical and medullary compartments of the ovarian structure (Figure 2A).

To further investigate the effects of TH on ovarian structure, Masson’s trichrome staining was performed. The results demonstrated a significant increase in fibrosis staining intensity in the ovaries of hyperthyroid rats compared to controls (Figure 2B). Notably, EGCG treatment reduced the fibrosis signal in a dose-dependent manner.

### 3.2. Effects of EGCG on Ovarian OS

To assess the effects of EGCG on ovarian OS in hyperthyroid rats, the levels of key antioxidant enzymes (CAT, T-SOD, and GSH-PX) and the lipid peroxidation end product MDA were measured.

**Figure 3:**
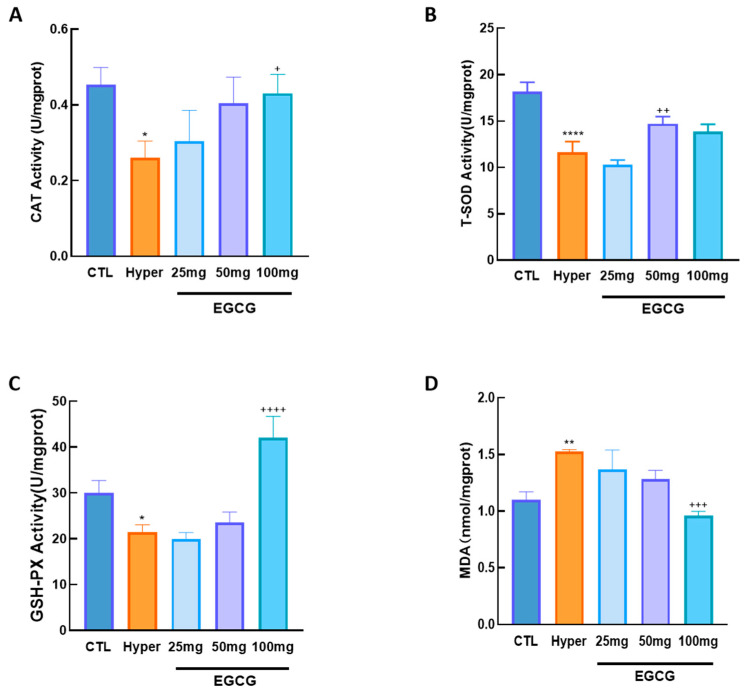
The effect of EGCG on oxidative stress in ovarian cells of hyperthyroid rats. The hyperthyroid rat model was established by intraperitoneal injection of L-thyroxine for 14 days, followed by EGCG treatment with intraperitoneal injections of 25, 50, and 100 mg/kg body weight. Total ovarian protein was extracted from each group of rats and the levels of antioxidant enzymes CAT (**A**), T-SOD (**B**), and GSH-PX (**C**), as well as the level of lipid peroxidation end product MDA (**D**) were detected. Data are presented as mean ± standard error of the mean of three independent experiments. * *p* < 0.05; ** *p* < 0.01; **** *p* < 0.0001, compared with CTL; + *p* < 0.05; ++ *p* < 0.01; +++ *p* < 0.001; ++++ *p* < 0.0001, compared with Hyper.

The results showed that the levels of antioxidant enzymes in hyperthyroid rat ovaries were significantly decreased, while MDA levels were markedly elevated. To investigate the effects of EGCG on OS, hyperthyroid rats were treated with varying concentrations of EGCG. Treatment with EGCG significantly upregulated the levels of antioxidant enzymes and concurrently reduced MDA levels (Figure 3).

### 3.3. Effects of EGCG on Ovarian ERS

It has been reported that ERS is closely linked to GC apoptosis. To investigate the effects of EGCG on ERS, the protein expression levels of GRP78, p-eIF2α, ATF4, CHOP, and Caspase-3 were analyzed using Western blotting.

**Figure 4:**
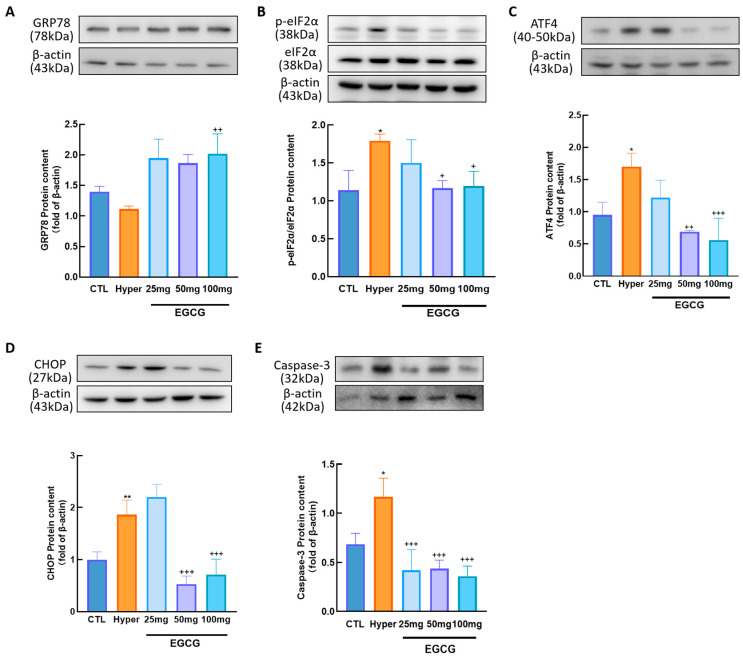
The effect of EGCG on endoplasmic reticulum stress in the ovaries of hyperthyroid rats. Extract total ovarian protein from each group of rats and detect the expression levels of endoplasmic reticulum stress-related proteins GRP78 (**A**), p-eIF2α (**B**), ATF (**C**), CHOP (**D**), and Caspase-3 (**E**). Data are presented as mean ± standard error of the mean of three independent experiments. * *p* < 0.05; ** *p* < 0.01, compared with CTL; + *p* < 0.05; ++ *p* < 0.01; +++ *p* < 0.001.

The results showed that GRP78 (Figure 4A) expression was decreased in hyperthyroid rat ovaries, whereas the levels of ERS-related proteins, including p-eIF2α (*p* < 0.05, Figure 4B), ATF4 (*p* < 0.05, Figure 4C), CHOP (*p* < 0.01, Figure 4D), and Caspase-3 (*p* < 0.05, Figure 4E), were all elevated. Notably, EGCG treatment reversed these changes in hyperthyroid ovaries.

### 3.4. Effect of T_3_ GCs Growth

**Figure 5:**
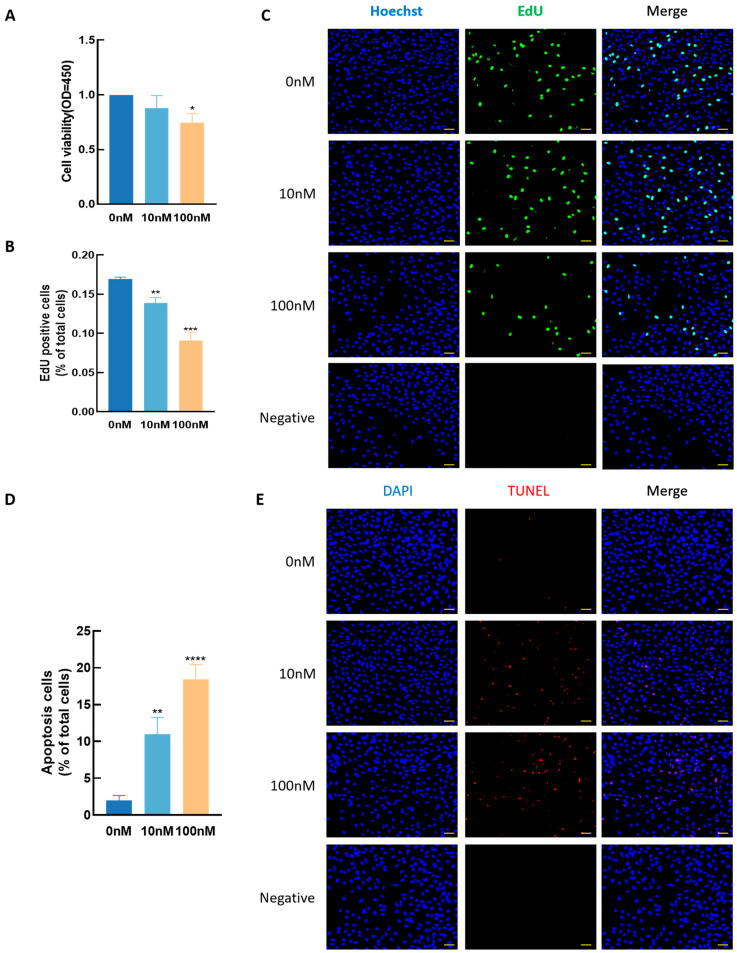
Effects of T_3_ on granulosa cells growth at higher concentration. The cell viability (**A**), cell proliferative (**B**,**C**) capacity and the apoptosis (**D**,**E**) of granulosa cells were detected after treatment with different concentrations of T_3_. The nucleus was stained with Hoechst (blue fluorescence) and EdU labeled as green fluorescence, scale bars = 50 μm. Data are presented as mean ± standard error of the mean of three independent experiments. * *p* < 0.05; **, *p* < 0.01; ***, *p* < 0.001, **** *p* < 0.0001, compared with the 0 nM group.

To investigate the effects of T_3_ on GC development, cells were treated with varying concentrations of T_3_ (0, 10, and 100 nM) for 48 h, and cell viability, proliferation, and apoptosis were assessed using CCK8, EdU, and TUNEL assays, respectively. As shown in Figure 5, higher concentrations of T_3_ significantly reduced cell viability (*p* < 0.05, Figure 5A) and proliferation (*p* < 0.01, Figure 5B,C) while simultaneously increasing apoptosis (*p* < 0.01, Figure 5D,E).

### 3.5. Effects of EGCG on GC Growth

**Figure 6:**
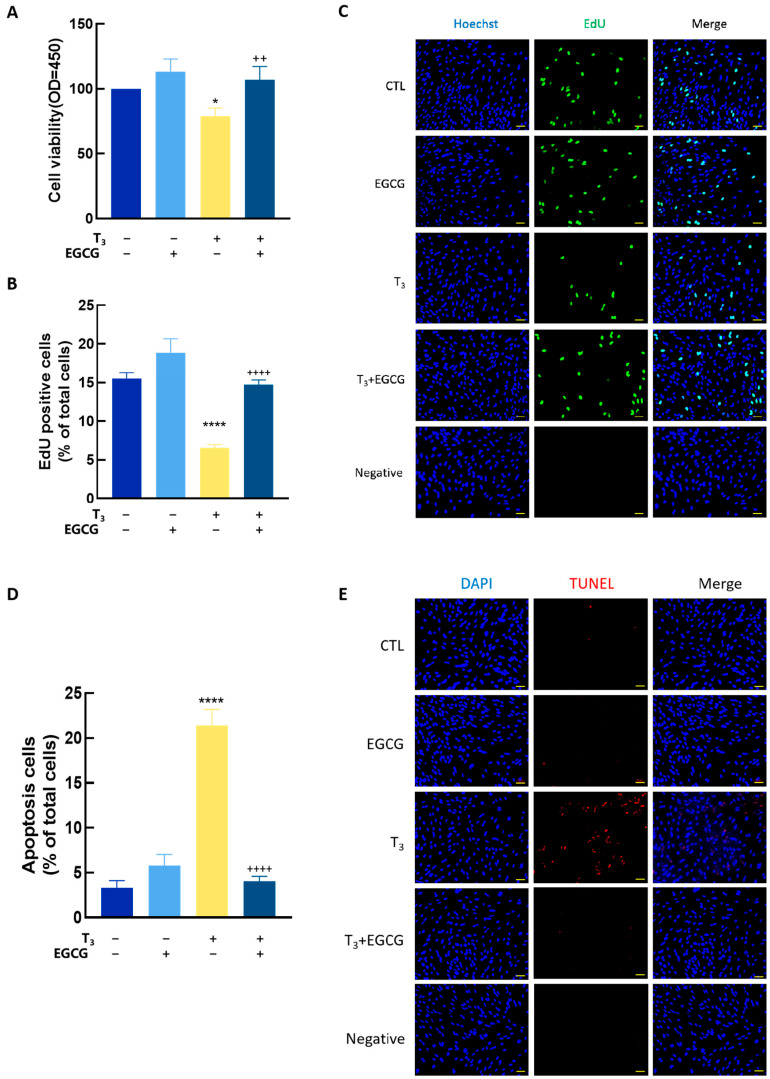
The effects of T_3_ and EGCG on granulosa cells growth. The cell viability (**A**), cell proliferative (**B**,**C**) capacity and the apoptosis (**D**,**E**) of granulosa cells were detected after treatment with T_3_ (100 nM) and/or EGCG for 48 h. The nucleus was stained with Hoechst (blue fluorescence) in EdU assay and EdU labeled as green fluorescence; the nucleus was stained with DAPI (blue fluorescence) in the TUNEL assay and TUNEL was labeled as red fluorescence, scale bars = 50 μm. Data are presented as mean ± standard error of the mean of three independent experiments. * *p* < 0.05; **** *p* < 0.0001, compared with CTL; ++ *p* < 0.01; ++++ *p* < 0.0001, compared with T_3_.

During follicular development, apoptosis of GCs is a major driver of follicular atresia, and the dysregulation of TH can induce abnormal GC development. Treatment with 100 nM T_3_ significantly reduced cell viability (*p* < 0.05, Figure 6A) and proliferation (*p* < 0.0001, Figure 6B,C), while higher T_3_ concentrations markedly increased apoptosis. Although EGCG alone had no significant effect on cell growth or apoptosis, it effectively reversed the detrimental effects of high-concentration T_3_ on GCs (*p* < 0.0001, Figure 6D,E).

### 3.6. EGCG Decreased T_3_-Induced ROS in GCs

**Figure 7:**
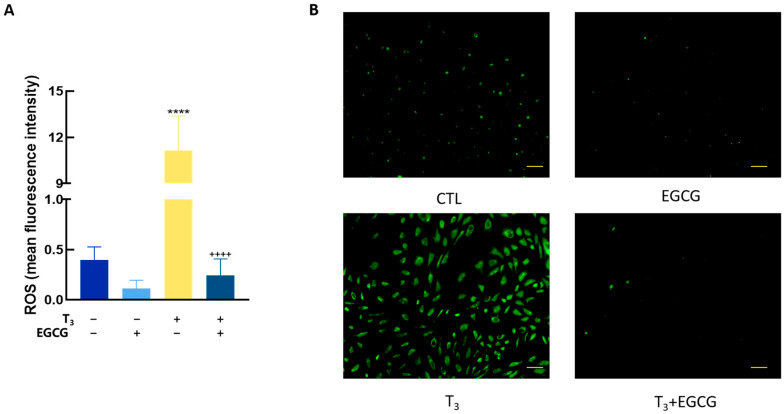
Effects of T_3_/EGCG on ROS in rat granulosa cells. Granulosa cells were co-treated with T_3_ (100 nM) and/or EGCG for 48 h and the ROS levels were measured. (**A**) bar chart showing relative ROS levels in GCs; (**B**) Fluorescence microscopy images of GCs stained with DCFH-DA (ROS labeled as green fluorescence), scale bars = 50 μm. Data were presented as mean ± standard error of the mean of three independent experiments. **** *p* < 0.0001 compared with CTL; ++++ *p* < 0.0001 compared with T_3_.

To investigate the effects of T_3_ and EGCG on OS in GCs, cells were co-treated with 100 nM T_3_ and EGCG for 48 h. ROS levels were assessed by measuring DCF fluorescence intensity. As shown in Figure 7, T_3_ treatment significantly increased ROS levels (*p* < 0.0001), whereas co-treatment with EGCG markedly attenuated this effect (*p* < 0.0001).

### 3.7. EGCG Decreased T_3_-Induced ERS in GCs

**Figure 8:**
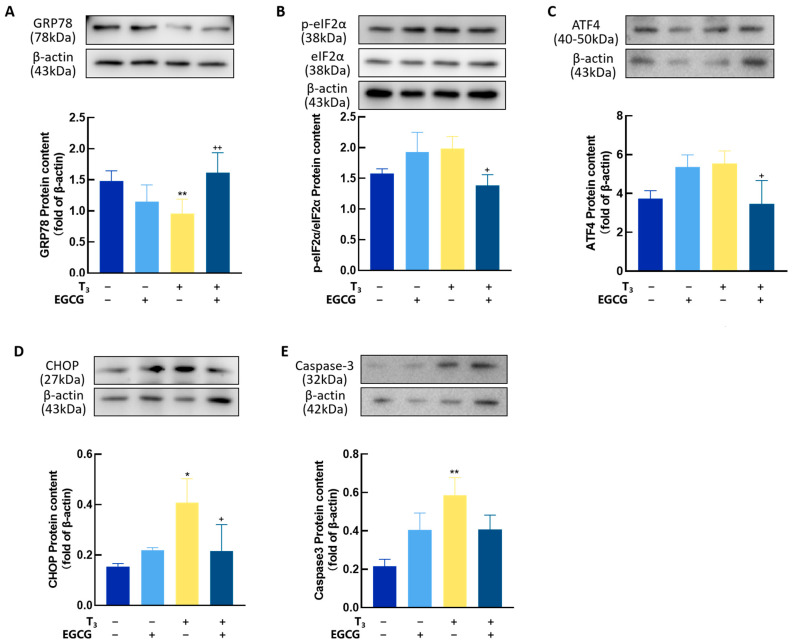
The effect of EGCG on endoplasmic reticulum stress in granulosa cells. Extract total protein from each group of granulosa cells and detect the expression levels of endoplasmic reticulum stress-related proteins GRP78 (**A**), p-eIF2α (**B**), ATF (**C**), CHOP (**D**), and Caspase-3 (**E**). “+” means the durg was added, “−” means not added. Data are presented as mean ± standard error of the mean of three independent experiments. * *p* < 0.05, compared with CTL, ** *p* < 0.01, compared with CTL; + *p* < 0.05; ++ *p* < 0.01; compared with T_3_.

To further evaluate the effect of EGCG on T_3_-induced ERS in GCs, the expression of ERS-related proteins, including GRP78, p-eIF2α, ATF4, CHOP, and Caspase-3, was analyzed (Figure 8). The results showed that T_3_ significantly reduced GRP78 expression (*p* < 0.01, Figure 8A), an effect that was mitigated by EGCG treatment. Additionally, T_3_-induced upregulation of p-eIF2α, ATF4, CHOP, and Caspase-3 was significantly reversed by EGCG. These findings indicate that EGCG can enhance the expression of the endoplasmic reticulum chaperone protein GRP78 and attenuate ERS in GCs.

## 4. Discussion

In the present study, we investigated the effects of TH and EGCG on ovarian function. Our results demonstrated that EGCG promotes ovarian cell development and protects against TH-induced damage in hyperthyroidism. Furthermore, EGCG alleviates T_3_-induced OS in GCs, exerting this cytoprotective effect through suppression of ROS-associated damage via the eIF2α/ATF4 signaling pathway. These findings suggest that EGCG may serve as a potential adjunct therapy for mitigating hyperthyroidism-associated female reproductive dysfunction.

The present results demonstrate that hyperthyroid rat ovaries exhibit disorganized tissue architecture, increased fibrosis, and elevated cellular apoptosis. Follicular development was also impaired, as evidenced by an increased number of atretic follicles and a decreased number of antral follicles, highlighting the adverse effects of hyperthyroidism on ovarian function. These findings are consistent with previous studies [26,27,28]. In particular, the pronounced fibrosis observed around tertiary follicles in hyperthyroid rats may reduce ovarian elasticity, hinder follicular rupture, and compromise normal ovulation.

During OS, excessive ROS are generated, which can induce oxidative reactions in biomacromolecules and severely impair normal tissue function. Overproduction of ROS damages lipids, proteins, and DNA. Specifically, hydroxyl radicals (-OH) react with purine and pyrimidine bases in DNA, damaging the deoxyribose backbone [29]. These permanent modifications serve as mutagenic markers and represent an initial step in carcinogenesis and aging. ROS also react with lipids containing carbon-carbon double bonds, particularly polyunsaturated fatty acids (PUFAs), producing cytotoxic lipid peroxidation products such as MDA. Excessive MDA accumulation compromises cell membrane integrity and permeability, leading to metabolic dysfunction and apoptosis [30]. Furthermore, ROS can directly oxidize side chains of amino acids such as lysine, arginine, proline, and threonine, resulting in protein carbonylation. Carbonylated proteins have been implicated in various human diseases, including Alzheimer’s disease, chronic lung disease, chronic renal failure, diabetes, and sepsis [31]. Disorders of TH levels are known to affect follicular development [32,33]. Previous studies have shown that hyperthyroidism disrupts the pro-oxidant/antioxidant balance, inducing OS in multiple organs, including the liver [32,33], kidney [34,35], brain [36], and testes [34,37]. However, ovarian OS under hyperthyroid conditions remains largely unexplored. In the present study, antioxidant enzyme levels were significantly reduced, and the lipid peroxidation product MDA was dramatically increased in the ovary, indicating that hyperthyroidism induces OS in ovarian tissue.

OS can induce oxidation of biological macromolecules, damaging their secondary and tertiary structures, which leads to proteome instability and the accumulation of unfolded proteins [11]. GRP78 plays a key role in refolding these unfolded proteins and activates downstream pathways to alleviate protein accumulation through the unfolded protein response (UPR) [13]. In hyperthyroid rats, GRP78 expression in the ovary was markedly decreased, resulting in unfolded protein overload and the initiation of ERS. Elevated levels of ERS marker proteins, p-eIF2α, ATF4, CHOP, and Caspase-3, indicate that ROS-mediated apoptosis is activated via the eIF2α/ATF4 signaling pathway. Notably, GRP78 exhibited a distinct expression pattern compared to other ERS markers. This biphasic response reflects the dynamic nature of ERS: GRP78 is transiently upregulated during initial stress to restore ER homeostasis, but its expression declines during prolonged or severe stress when apoptosis predominates [38,39]. These findings are consistent with our previous studies [40]. EGCG, the primary bioactive polyphenol in green tea, possesses multiple phenolic hydroxyl groups that confer potent antioxidant properties [41]. Its regulatory effects may involve directly scavenging free radicals, thereby reducing MDA levels, as well as upregulating antioxidant enzyme activity, ultimately alleviating ovarian OS [42,43]. Our results confirmed that EGCG treatment effectively mitigated OS in hyperthyroid rat ovaries.

Furthermore, multiple studies have demonstrated that EGCG can modulate ERS by downregulating marker proteins such as GRP78, CHOP, and Caspase-3 [44,45,46]. In the present study, treatment with 100 mg/kg EGCG significantly reduced the expression of p-eIF2α, ATF4, CHOP, and Caspase-3, confirming its efficacy in alleviating hyperthyroidism-induced ovarian ERS and suppressing apoptosis.

To further investigate the effects of THs and EGCG on follicular development, GCs were treated with EGCG. Since T_3_ is the primary active form of TH in in vivo experiments, administered T_4_ can be converted to T_3_ by deiodinases; however, in in vitro assays, where deiodinase activity is absent, T_3_ was used directly. The results showed that higher concentrations of T_3_ markedly decreased cell viability and proliferation while increasing apoptosis. These detrimental effects were effectively reversed by EGCG treatment. T_3_ exposure also caused an abnormal elevation of ROS levels in GCs, whereas co-treatment with EGCG significantly attenuated this OS response. The protective effect of EGCG against apoptosis may be attributed to decreased ROS production, achieved by directly scavenging free radicals, reducing MDA levels, and upregulating antioxidant enzymes. Moreover, T_3_-induced ROS overproduction activated ERS, promoting the upregulation of apoptotic executors via the ERS-mediated pathway. These findings indicate that excessive TH can impair follicular development through both OS and ERS. It should be noted that the T_3_ concentration (100 nM) used in our in vitro experiments is supraphysiological; this higher concentration was intentionally selected to establish a robust model of extreme TH exposure, allowing for a clearer elucidation of the potential mechanisms underlying follicular damage under severe hyperthyroid conditions.

EGCG administration significantly downregulated ERS markers, demonstrating its effectiveness in alleviating hyperthyroidism-induced ovarian ERS. These findings indicate that EGCG can also mitigate OS in hyperthyroid ovaries, highlighting its potential as a therapeutic agent.

Although EGCG effectively scavenges ROS and mitigates hyperthyroidism-induced ovarian damage at appropriate concentrations, excessive intake can cause hepatotoxicity. Studies have shown that a single oral administration of 1500 mg/kg EGCG in male CF-1 mice significantly increased plasma alanine aminotransferase (ALT) levels and mortality [47]. This hepatotoxicity is attributed to the pyrogallol structure on EGCG’s B-ring, which generates ROS through auto-oxidation and subsequently induces apoptosis. Because EGCG is primarily metabolized in the liver, this organ is particularly susceptible to toxicity. In the present study, rat mortality occurred within two days of EGCG administration at 200 mg/kg, consistent with previous reports of lethal effects at the same dose in Kunming mice [48]. Therefore, EGCG must be administered within a safe dosage range, with the optimal dose in this study determined to be 100 mg/kg to avoid hepatic injury. These findings demonstrate that the antioxidant properties of EGCG can effectively alleviate both OS and ERS in ovaries under hyperthyroid conditions, highlighting its potential as a therapeutic agent.

## 5. Conclusions

In conclusion, our findings demonstrate that excessive TH impairs ovarian development through ROS-mediated OS and subsequent ERS via activation of the phosphorylated eIF2α (p-eIF2α)/ATF4 pathway. EGCG effectively attenuated these pathological processes by scavenging ROS, mitigating OS, and alleviating ERS, ultimately enhancing cell viability and suppressing apoptosis to improve TH-induced ovarian dysfunction. These results provide a mechanistic basis for considering green tea-derived EGCG as a potential therapeutic adjuvant for managing hyperthyroidism-associated female reproductive disorders.

## Data Availability

All data generated or analyzed during this study are available from the corresponding author upon reasonable request.
